# Temporal Structure in Haptic Signaling Under a Cooperative Task

**DOI:** 10.3389/fnhum.2019.00372

**Published:** 2019-11-27

**Authors:** Nicolas Thorne, Juliane J. Honisch, Toshiyuki Kondo, Slawomir Nasuto, Yoshikatsu Hayashi

**Affiliations:** ^1^Division of Biomedical Sciences and Biomedical Engineering, School of Biological Sciences, University of Reading, Reading, United Kingdom; ^2^School of Psychology and Clinical Language Sciences, University of Reading, Reading, United Kingdom; ^3^Department of Computer and Information Sciences, Tokyo University of Agriculture and Technology, Koganei, Tokyo, Japan

**Keywords:** haptic (tactile) perception, proto-language, Tsallis entropy, joint action, cooperative task, long range correlations, temporal correlations

## Abstract

Haptic communication between humans plays an important role in society. Although this form of communication is ubiquitous at all levels of society and of human development, little is known about how synchronized coordination of motion between two persons leads to higher-order cognitive functions used in communication. In this study, we developed a novel experimental paradigm of a coin-collecting task in which participants used their hands to control a rod to jointly collect the coins on the screen. We characterized the haptic interactions between paired participants while they were taking part in a cooperative task. The individual participants first completed this task on their own and then with a randomly assigned partner for the cooperative task. Single participant experiments were used as a baseline to compare results of the paired participants. Forces applied to the rod were translated to four possible haptic states which encode the combination of the haptic interactions. As a next step, pairs of consecutive haptic states were then combined into 16 possible haptic signals which were classified in terms of their temporal patterns using a Tsallis q-exponential function. For paired participants, 80% of the haptic signals could be fit by the Tsallis q-exponential. On the other hand, only 30% of the signals found in the single-participant trials could be fit by the Tsallis q-exponential. This shows a clear difference in the temporal structures of haptic signals when participants are interacting with each other and when they are not. We also found a large difference in the number of haptic signals used by paired participants and singles. Single participants only used 1/4 of the possible haptic signals. Paired participants, on the other hand, used more than half of the possible signals. These results suggest that temporal structures present in haptic communication could be linked to the emergence of language at an evolutionary level.

## 1. Introduction

For social animals, moving bodies in a coordinated manner plays an important role in facilitating social interactions. Such coordinated actions are common in human group activities such as playing music, dancing and jointly carrying objects (Hayashi and Kondo, 2013; Hayashi and Sawada, 2013; Codrons et al., 2014; Wing et al., 2014). Social interactions rely on the exchange of information to identify a shared objective and create an action-perception loop between the two individuals.

To study the social interactions that take place between individuals to coordinate their bodies in space and time, we need to move away from traditionally held assumptions which say that high- level cognitive processes can be studied in isolation from the participant's natural environment. Instead, we must study how the mind and body are embedded in the world (Van Dijk et al., 2008). Embodied dynamics focuses on self-organizing dynamic systems and claims that cognitive processes emerge from the closed loop of continuous sensory-motor interactions involving the brain, body and environment (Varela et al., 2018). The central idea is to look at the mind as an embodied-dynamics system in the changing world. Using a Human-Computer Interface can overcome practical difficulties of studying complex human-to-human interactions in real time, reducing the parameters of social expression. For example, using the cursor motion and objects on a display, Auvray et al. developed the Perceptual Crossing Experiments (PCE) in which participants were asked to move an avatar and identify the other active player's avatar (Auvray and Rohde, 2012). Using the PCE, Froese et al. found that paired participants simultaneously click to make a judgment of interactions with the other, rather than clicks in isolation, indicating that social judgments were not so much based on an individual recognition of the other, but rather on a mutually shared recognition of each other, i.e., on an interactively shared cognitive process (Froese et al., 2014). This finding is strong evidence of an irreducibly collective mode called the “we-mode” for interacting agents of sharing minds (Gallotti and Frith, 2013).

In the case of physical human-human interactions, the haptic sensory feedback between each participant can become a channel used for the mutual sharing of intentions. This channel plays a primary role in the construction of a shared motor plan to achieve a common goal. Van der Wel et al. asked paired participants to move an upside-down pendulum rhythmically between two targets (van der Wel et al., 2011). Participants controlled the pendulum by pulling on cords attached to the bottom of the pole. Van der Wel found that paired participants produced more overlapping forces than single participants. These results suggested that paired participants used the overlapping forces as a haptic channel which was used to transfer haptic information and facilitate coordination between them.

Many experiments with paired participants have shown improvements in task performance when individuals were weakly coupled to each other. In one such experiment performed by Ganesh et al. two participants taking part in the same task were weakly connected by a virtual elastic band to another participant. Participants were not consciously aware of their connection to another person. They subconsciously used information transmitted through the virtual elastic band to enhance their performance. Participants achieved noticeably better results in the task when working with a partner than they did working alone (Ganesh et al., 2014). Work done by Zenzeri et al. also showed an increase in performance in a bi-manual stabilization task when naive participants trained with an expert as a partner vs. training with another naive participant. Although expert-naive groups showed a smaller effort index than naive-naive groups from the beginning of the training, only the naive-naive groups showed any reductions in effort during the experiment (Zenzeri et al., 2011). They first hypothesized that it was important for naive participants to properly explore the space to learn a complete representation of the dynamics of the task (Ḿıreles et al., 2016; Mireles et al., 2017; Galofaro et al., 2017).

Even though haptic communication has been studied in the context of skill transfer, the main focus has been the characterization of motor performance as a result of haptic communication. Thus, the question still arises: What mechanisms allow partners in collaborative tasks to exchange and interpret haptic signals? This haptic communication is done through the action-perception loops in which the participants move their hands and feel the force transmitted by their partner. More crucially, the key to this haptic communication is to couple the action-perception loops between the paired participants, i.e., one sends the haptic signal to the other, the other receives the haptic signal, processes information and sends back the haptic signal, and the next action-perception loop continues.

This kind of coupling of the action-perception loops through haptic interactions should be characterized as a function of time; thus, we aim to reveal the temporal structure of the haptic communication.

The paired participants, while jointly lifting and moving an object, generated haptic channels that they used to communicate with each other (Sebanz et al., 2006; van der Wel et al., 2011). We hypothesize that this haptic channel is used to transmit bi-directional haptic signals carrying information about the participant's next target on the display. The analysis presented in this paper focuses on characterizing the temporal structures of haptic signals that naturally emerge from the haptic interactions. Those temporal structures might be related to proto-language studies. For example, research into emergent proto-languages by Uno et al. showed that participants would communicate using a combination of two modes, i.e., participants either interpreted the presented pattern literally (dynamical mode) or created a narrative around the pattern (metaphorical mode) (Uno et al., 2011). The dynamical mode is characterized by larger hamming distances, meaning that more elements of the pattern change in the dynamical mode. This mode also shows a lower linearity value, showing that there is a larger chance for patterns to repeat. The metaphorical mode is characterized by shorter hamming distances; this means that fewer of the elements of the pattern are changed in each turn. This mode shows larger linearity meaning that patterns are rarely repeated.

In order to study the temporal correlations, present in haptic communication between paired participants, we developed a joint coin-collecting paradigm. In this paradigm, participants jointly controlled a plastic cylinder to collect coins on a computer display. We characterized the force-force interactions for both single and paired participants and analyzed their temporal patterns in order to extract features of haptic signaling between paired participants in a cooperative task.

## 2. Experimental Protocol

### 2.1. Subjects

In total, 30 participants and 15 paired participants took part in the experiment, one pair encountered technical issues during the experiment and had to be removed. They were all recruited by the recruitment system of the University of Reading, were all right handed and between the ages of 18 and 35. Participants first completed the experiment alone and came back a few days later to complete it as paired participants. For paired experiments, participants were selected so that the two members had not met each other before the experiment and were of the same sex. The experiment was granted ethical approval by the University of Reading. All participants gave their informed written consent to participate in the experiment and have their data used for publication.

### 2.2. Joint Coin-Collecting Paradigm

**Figure 1:**
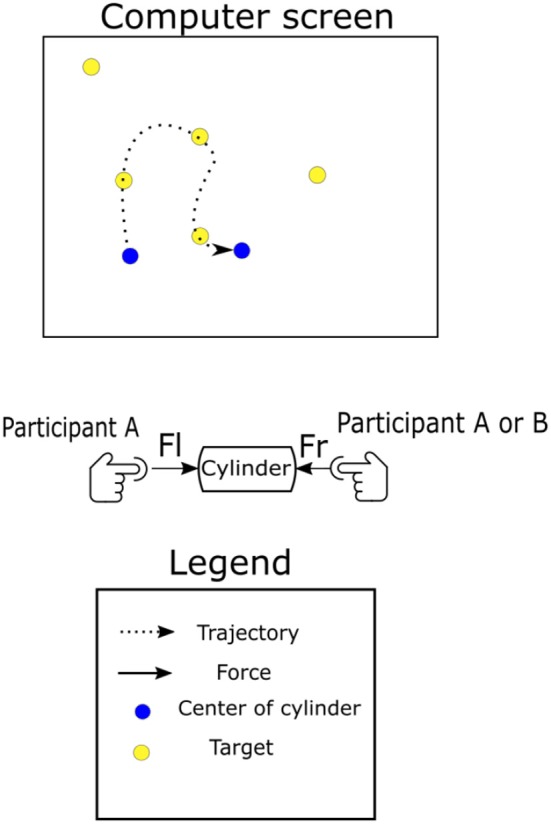
Illustration of the paradigm developed for this experiment. Participants are shown either 1, 2, 5, or 10 yellow targets on the computer screen at a time. When one is touched by the blue tracer, the target will disappear and a new one will appear at a random location. The game can be played by one or two participants at a time; in the case of paired participants, participant A was seated on the right side and use his/her right index finger. Participant B was on the left side and use his/her left index finger.

The experiment consisted of a modified target reaching task, which we named the "joint coin-collecting" task. The task could be completed either by a single participant as a bi-manual task or by the paired participants. Participants moved in virtual 2D space using a peripheral device constructed using a 3D-printed rod which was attached to the end effector of the haptic device as shown in Figure 1. During the coin-collecting task, participants followed the points below to jointly control the cylinder;

1. To lift the cylinder, both participants must place their index fingers on the plastic dome on either end of the cylinder. When the task is done by single participants, they must place one index finger on each side of the cylinder, thus making the task bi-manual. When the task is done as paired participants, participant A will place his/her right index finger and participant B will use his/her left index finger.
2. They must place a minimum horizontal force on the device to create enough friction so that they can lift it off the tabletop. This means that both participants share control over the horizontal and vertical positions of the cylinder.
3. A purely vertical movement would have a net force of 0 within our experimental setup, and horizontal movements are made possible by modulating the force on either side of the cylinder and creating a net negative or positive force.
4. For horizontal movements, the net force on the cylinder will need to be either positive or negative, depending on the direction.

Note here that to restrict the number of behavioral cues available to the participants, the apparatus did not allow for any tilting or twisting of the rod.

All participants were instructed to collect as many yellow targets, “coins,” as possible in 40 seconds. There were four possible conditions which were designed to increase the difficulty of choosing the next target under cooperation; One target, Two targets, Five targets, Ten targets.

Pairs were asked to refrain from verbal communication during the course of the experiment and maintain their focus on the screen. This restricted participants to communicate via the forces used to move a cylinder. Those forces were measured using an FSR-400 Interlink Electronics force-sensing resistor (FSR) attached at both ends of the cylinder. A soft plastic dome of the same diameter was placed on top of the FSR sensor to evenly distribute the force from the participant's finger to the surface of the sensor. These sensors could only record forces along the horizontal axis, thus our method only takes into account haptic signals in the horizontal axis. Acceleration was measured using a 3-axis accelerometer (AdaFruit ADXL335) attached to the center of the cylinder.

Participants were seated at a comfortable distance from the haptic arm and asked to pick up and move the cylinder by placing one index finger on each plastic dome.

In the case of paired participants, the participant on the left-hand side of the computer used their left index finger while participants on the right-hand side of the computer used their right index finger. During each trial, participants were given 40 s to collect as many yellow targets as possible by controlling a blue joint cursor on the display. There were four randomly assigned conditions, each corresponding to a different number of coins on the display: 1, 2, 5, and 10 coins. When a coin was touched by the cursor, a new coin was placed at a random location on the display, keeping the number of coins on the display constant. Each condition was repeated 10 times for a total of 400 s of recorded activity. The entire experiment lasted 1,600 s (27 minutes).

## 3. Analysis of Experimental Data

### 3.1. Defining Haptic States

All participants successfully coordinated their movements and collected coins during the task period. We hypothesized that to correctly synchronize their movements, paired participants had to exchange information about their next target and form a joint motor plan to reach their target without dropping the device. Van der Wel et al. identified a possible means of communication between participants that may be responsible for the participants' ability to carry out this experiment (van der Wel et al., 2011). They proposed that when paired participants apply a force on the device at the same time, a haptic channel is created. In this study, we extended the concept of a haptic channel (Sebanz et al., 2006; van der Wel et al., 2011) by using the forces exerted by paired participants to identify what we call haptic states. Haptic states are defined as combinations of binary force applied at both sides of the cylinder. There are four possible haptic states, listed below. The item number is the state label and the description shows the binary values for both forces.

1. No forces are above the threshold, *F*_*a*_ = 0, *F*_*b*_ = 0.
2. Only participant A is applying an above-threshold force on the device, *F*_*a*_ = 1, *F*_*b*_ = 0.
3. Only participant B is applying a force that is above the threshold, *F*_*a*_ = 0, *F*_*b*_ = 1.
4. Both forces are above the threshold, *F*_*a*_ = 1, *F*_*b*_ = 1.

**Figure 2:**
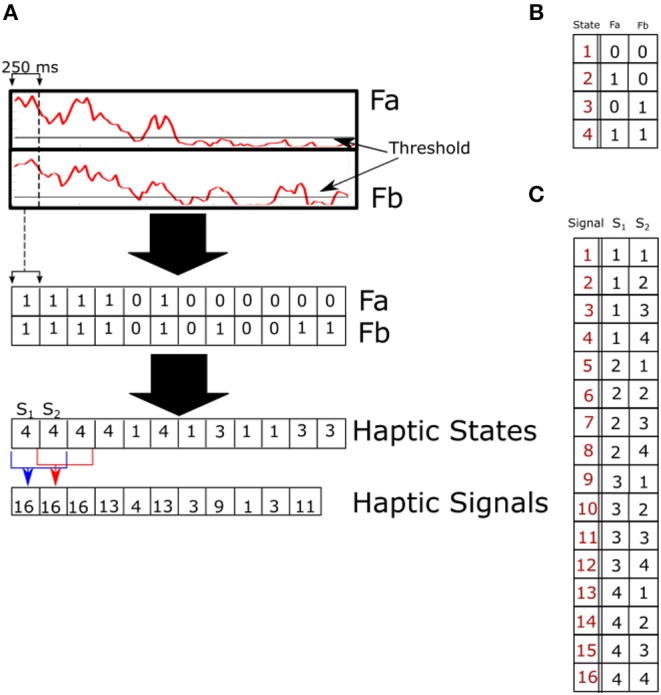
**(A)** Method used to binarize the continuous forces recorded from force sensors **(A,B)**. Forces are first binarized using a threshold, and this results in a binary time series for each force sensor. Binary signals are then combined to form a single time series of haptic states which encodes both streams of information. To uncover the signaling being used by paired participants to communicate with each other, we further combined haptic states into haptic signals using a sliding window. Signals of different lengths were tested with haptic signals of length two giving the highest non-extensivity scores. **(B)** Labels of haptic states comprised of two binarized forces **(A,B)**. The resulting haptic state is shown in red. **(C)** Haptic signals comprised of two consecutive haptic signals. Haptic signal numbers are marked in red, states at time t, and t+1 are marked as *S*_1_ and *S*_2_, respectively.

A diagram showing the procedure to define the haptic states is given in Figure 2.

Force data was first low-pass filtered using a Butterworth filter (Matlab zero-phase filter function) with a 25-Hz cutoff frequency. The first and last seconds of each trial were discarded to remove any noise caused by picking up and putting down the cylinder. Data collected from force sensors were first normalized by calculating the z-score for each trial,

(1) $$\begin{array}{r} F_{N}=\frac{F\left(t\right)-\mu F}{\sigma F}, \end{array}$$

where *F*_*N*_ is the normalized force, *F*(*t*) is the force time series, μ*F* is the average force, σ*F* is the standard deviation of the force and *F*_*t*_ is the threshold. Binarized forces were calculated by comparing the average value of a 250-ms time window to 10% of the maximum value of the normalized forces for a particular pair or single. This was chosen so that the resulting threshold was consistently above the noise floor of the sensor and still able to identify perceptible changes during haptic interactions.

Any value larger than the threshold was recorded as a 1, and anything below the threshold was recorded as a 0:

(2) $$B(t)=\{\begin{array}{cc} 0 & \sum_{i=1}^{250}\frac{F_{N}}{250}<F_{t}\\ 1 & \sum_{i=1}^{250}\frac{F_{N}}{250}\geq F_{t} \end{array}$$

An example of this process is shown in Figure 2A. The binarized forces were then combined to create a single time series which encodes both forces A and B as shown in Figure 2A.

### 3.2. Haptic Signals

When analyzing forms of communication between participants, it is necessary to define a time window that corresponds to the participants' window of attention. The haptic states within the time windows make up the haptic signals that paired participants used as a means of exchanging information with each other. Defining this window of attention is vital for analyzing the temporal dependencies of the haptic signals. The upper bound for the window length was informed by research looking at the optimal presentation rate of haptic cues. Tan et al. presented a target haptic cue sandwiched between two masks which were also haptic cues (Tan et al., 1999). In their experiments, participants were asked to correctly identify the target cue. Haptic cues were a combination of single, double and triple frequency waveforms presented to one of three fingers. By analyzing the percentage of correct identifications as a function of the time before the target was presented, they concluded that the optimal presentation rate was about 2.2–3.0 cues per second.

To stay within this time window, haptic states were calculated using a 250-ms window in our study, and haptic signals were defined as two consecutive haptic states. A list of haptic signals which consists of two haptic states and their corresponding states with signal numbers are given in Figures 2B,C. With this definition, we can begin to investigate the temporal structure that emerges during haptic interactions and compare them with the two modes of communication identified by the above-described proto-language study (Uno et al., 2011).

### 3.3. Calculating Non-extensivity Measure for Haptic Signals

To analyze the temporal structures of haptic signals, we applied a generalized Boltzmann-Gibbs (BG) distribution called the Tsallis distribution, which is described by Equations 4 (Appendix) and 5 (Appendix) (Tsallis, 1988, 1999; Tsallis and Brigatti, 2004). Tsallis' generalization allows for the characterization of systems not properly described by BG such as ones with long-range interactions. By fitting Equation 9 (Appendix) to the distribution of the collected data we can calculate the *q* value associated with it.

Long and short-range temporal correlations refer to the shape of the distribution of distances between consecutive entries of a given haptic signal. As the distribution becomes an exponential distribution where there is no long tail, the *q* value, known as the non-extensivity measure [shown in Equation 5 (Appendix)], approaches 1. On the other hand, as the distribution approaches a power law, and gains a fatter tail, the *q* value is >1. Thus, the *q* value allows us to characterize different statistical distributions with a single number. Exponential distributions represents the timing of a series of events as a Poisson process where the occurrence of individual events is independent. The power law distribution, on the other hand, describes processes where past events influence future events, indicating that there is some correlation between the two events. In our analysis, we use a normalized q value, Δq (Equation. 3), which has been normalized to account for a random distribution of the haptic signal whose Δ*q* value is being calculated. We refer to larger Δ*q* values as having more long-range correlations because of the fatter tail in the distribution of distances between successive appearances of a signal. This means that signals with larger Δ*q* values have a wider spectrum of temporal correlations. The structure of these temporal correlations is what participants use to facilitate their haptic communication while achieving a common goal.

This property of the Tsallis distribution has been used to characterize the spatial and temporal patterns in complex systems such as earthquakes. Works done by Abe and Suzuki have successfully detailed new laws relating to both the spatial and temporal distances between earthquake epicenters (Abe and Suzuki, 2002, 2003). They found that *q* values smaller than 1 indicated shorter range spatial correlations between earthquakes, these are related to the aftershocks which accompany most earthquakes. On the other hand, *q* values larger than 1 characterized the longer-range temporal correlations found in earthquake epicenter time series. Long-range temporal correlations were previously found to be properly described by the Zipf-Mandelbrot law (Abe and Suzuki, 2002). Through this research Abe et al. concluded that the non-extensivity measure was an appropriate and powerful way of characterizing phenomena with different ranges of correlations.

The non-extensivity measure has also been used as a method of identifying important sequences of symbols such as important words in a text (Jamaati and Mehri, 2017) or functional DNA (Moghaddasi et al., 2017). Functional is used to distinguish sections of DNA that are critical for development. For example, Moghaddasi et al. found that the CG dinucleotide has the highest clustering level and is also biologically important given that it is associated with methylation, which is essential for normal development.

**Figure 3:**
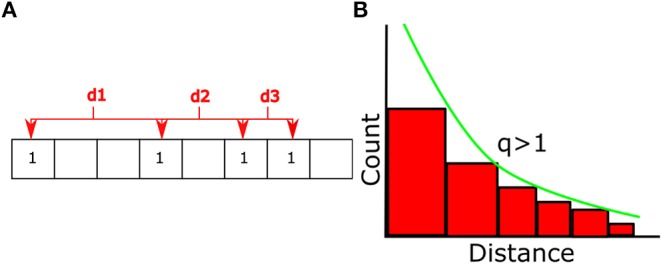
**(A)** Distances between all consecutive entries of a particular signal are recorded. The distribution of these distances will then be fit by the q-exponential detailed in Equation 9 (Appendix). **(B)** Fit of q-exponential to distribution of distances. *q* values larger than 1 are similar to power laws. Surrogate data sets are created by randomizing the positions of each haptic signal and creating a distribution of the new distances as detailed in Equation 3.

Temporal distances between two successive signals of the same type were calculated using the method shown in Figure 3A, *d* = *t*_*i*+1_−*t*_*i*_ for all signals present in the data. By fitting Equation 9 (Appendix) to the cumulative distribution of *d*, we calculated the non-extensivity measure *q* for each signal during each trial. To reduce the influence of noise on the results, *q* values were normalized by creating random permutations of the original haptic signal time series. Distances between consecutive entries of each haptic signal and *q* values were then calculated the same way as with the original time series to create a set of surrogate *q* values. This procedure was repeated N times for each trial and averaged together as shown in Equation 3.

(3) $$\begin{array}{r} \Delta q=q_{d}-\left(\frac{1}{N}\overset{N}{\underset{i=1}{\sum }}q_{s}^{i}\right) \end{array}$$

where *q*_*d*_ is the *q* value calculated from the original data and $q_{s}^{i}$ is the *i*'th surrogate *q* value.

Positive values correspond to signals that have more long-range temporal correlations than the same number of events distributed randomly in time, while negative values correspond to signals with fewer long-range temporal correlations than the same number of events randomly distributed in time.

To identify the length of the time window of attention for the haptic signaling taking place between paired participants, we tested three different lengths of haptic signals. Haptic signals were created by combining a predetermined number of consecutive haptic states which correspond to the window of attention. These are encoded with a new number which we called the haptic signals. The window of attention was advanced one position for each haptic signal. We analyzed signals made up of one, two and three successive haptic states. Signals comprised of two states (also referred to as signals of length two) gave the best results and fit into the time scale reported by Tan et al. (1999). An example of this process can be seen in Figure 2, and a full table of all 16 haptic signals that were further analyzed is shown in Figure 2C.

Positions of each entry of a haptic signal with *m* total entries can be denoted as *t*_1_, *t*_2_…*t*_*m*_. Distances between two successive entries of a signal are calculated as *d*_*i*_ = *t*_*i*+1_−*t*_*i*_. By fitting q-exponential, described in the Equation 9 (Appendix) to the cumulative distribution function of distances for a particular signal, we can calculate the non-extensivity measure for that signal as shown in Figure 3B. Any signals that appeared fewer than 10 times were discarded and not fit with the q-exponential.

To be able to contrast findings from different participants, it was important to discard any undesired effect on the *q* value from the frequency of a particular signal. We corrected this by shuffling the haptic signal in time and calculating the new *q* value; this was done 500 times for each signal and the average is taken as the random *q* value for that signal. Δ*q* is then calculated as $q_{d}^{i}-<q_{r}^{i}>$ where $q_{d}^{i}$ is the *q* value calculated from the collected data for signal *i* and $<q_{r}^{i}>$ is the average of the randomized *q* values for signal *i*.

## 4. Results and Discussion

In our experimental paradigm, participants coordinated their movements to jointly lift and move a cylindrical rod. Using this peripheral device, participants were asked to play a simple coin-collecting game on the computer once alone as a bi-manual task and another time as paired participants.

**Figure 4:**
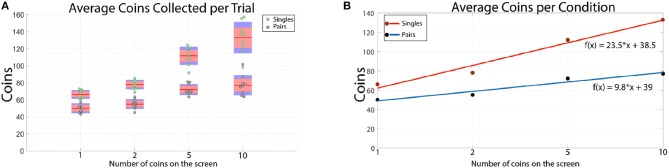
**(A)** The average number of coins per trial collected by all single and paired participants. Each data point corresponds to the average over all trials for a given single or pair. The red line shows the average of the individual data points. The red outline shows the 95% confidence interval of the average value and the blue outline shows the standard deviation. **(B)** The points represent the overall average number of coins collected for a given condition. These correspond to the red bars in **(A)**. The number of coins collected by single participants rises sharply as the number of coins on the screen increases. On the other hand, paired participants have a much slower increase in the number of coins collected during a 40 s trial. The equations given in the figure are the lines of best fit.

The average number of coins collected during each trial was consistently higher for single than for paired participants (Figure 4A). By fitting a linear curve to the averages, we found that the average number of coins collected by single participants increases twice as fast as the average collected by paired participants (Figure 4B). Single participants collected between 1.6 and 3.25 coins per second, while paired participants collected between 1.25 and 2 coins per second. This suggests that single participants were able to adapt to the task quicker than paired participants.

During the experiment, participants were able to freely adapt the available cognitive strategy. However, physical constraints coming from the haptic device (rod and robotic device), as explained in 2.2, naturally limited these strategies by requiring paired participants to share control of the cylinder in the horizontal and vertical movements.

During horizontal movements, one participant must apply force and take control over the device, while the other must be compliant and allow movement in the direction of the next target. For example, movements to the left of the display would be first initiated by the participant on the right-hand side, and the participant on the left-hand side would need to apply enough pressure to keep the device stable but be compliant enough to allow the cylinder to move horizontally. On the other hand, vertical movements require both participants to apply a small horizontal force to stabilize the cylinder. In summary, haptic communication in the form of the strategic movements was best characterized by the net horizontal force (*F*_*N*_ = *F*_*b*_−*F*_*a*_) to accelerate the cylinder in the direction of the next target. Although there seems to be no specific strategy in terms of path planning that participants might have developed during the experiment, we were able to observe that the paired participants did not collect the closest target from the current position of the joint tracer; data from both single and paired participants showed that on average there was only a 20% chance of choosing the closest target in the 10-coin condition, 70% for the 5-coin condition and 50% for the two-coin condition. These results suggest the emergence of a more complex strategy in haptic interactions, namely force-to-force interactions, as shown in the temporal structures in haptic states and signals.

**Figure 5:**
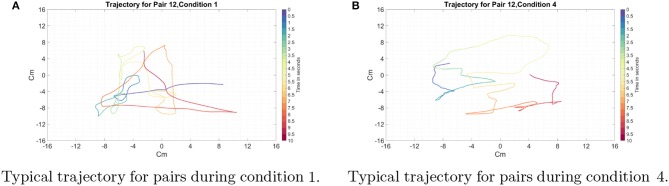
Typical example of the trajectory for pair 12 in the one and ten coin conditions. Each color represents a 500-ms time window, the beginning of which is labeled in the legend. Time 0 in both figures is the beginning of the trial. **(A)** Condition 1: Only one coin is present on the screen. **(B)** Condition 4: Ten coins are present on the screen. We found that the participants, despite the distinct nature of horizontal and vertical movements of the cylinder, showed smooth trajectories on the display with the velocity vectors in all directions.

Typical trajectories are shown in Figures 5A,B. We found that despite the distinct nature of the horizontal and vertical movements, participants showed smooth movements with the velocity vectors in all directions, rather than sequentially performing horizontal and vertical movements. This indicates that our participants had to cooperate in order to control movement in both the horizontal and vertical planes. The smoothness of these plots shows that participants were reliably able to coordinate their movements and explore the 2D space.

**Figure 6:**
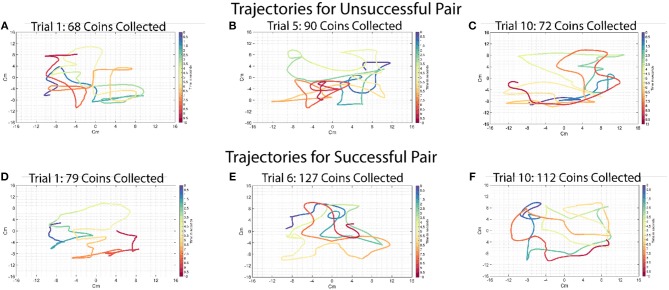
Typical example of trajectories for a successful and an unsuccessful pair in the ten-coin condition. Each color represents a 500-ms time window, the beginning of which is labeled in the legend. Time 0 in both figures is the beginning of the trial. The trajectories for both successful and unsuccessful pairs are visually similar and show no difference in movement smoothness. **(A)** This trajectory is taken from the first trial of the 10-coin condition of an unsuccessful pair. The pair was able to collect 68 coins in the 40 s they were given for the trial. This pair collected 63.8 coins on average during the 10-coin condition. **(B)** This trajectory is taken from the fifth trial of the ten-coin condition of an unsuccessful pair. The pair was able to collect 90 coins in the 40 s they were given for the trial. **(C)** This trajectory is taken from the tenth trial of the ten-coin condition of an unsuccessful pair. The pair was able to collect 72 coins in the 40 s they were given for the trial. **(D)** This trajectory is taken from the first trial of the ten-coin condition of a successful pair. The pair was able to collect 79 coins in the 40 s they were given for the trial. This pair collected 105.8 coins on average during the 10-coin condition. **(E)** This trajectory is taken from the fifth trial of the ten-coin condition of a successful pair. The pair was able to collect 127 coins in the 40 s they were given for the trial. **(F)** This trajectory is taken from the tenth trial of the ten-coin condition of a successful pair. The pair was able to collect 112 coins in the 40 s they were given for the trial.

**Figure 7:**
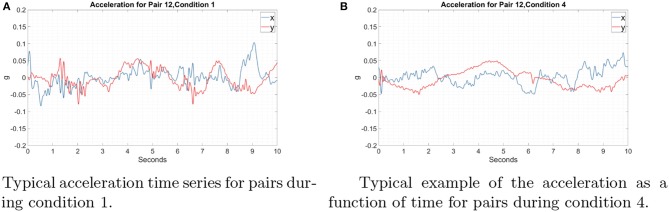
Typical example of acceleration as a function of time for pair 12 in the one and ten coin conditions. The blue trace represents the acceleration along the x-axis and the red the acceleration along the y-axis. Time 0 in both figures is the beginning of the trial. **(A)** Condition1: Only one coin was present on the screen. **(B)** Condition 4: Ten coins were present on the screen. Participants accelerated in both the x and y directions simultaneously, resulting in the velocity vectors in all directions.

Figure 6 shows some sample trajectories from successful (Figures 6A–C) and unsuccessful (Figures 6D–F) pairs. Figures 7A,B shows that acceleration along the x-axis and y-axis can happen at the same time to collect coins in all directions. These results show that simple coordination strategies such as division of labor, collecting the closest coin and maximization of movement smoothness were not being implemented. In the absence of any other form of communication, force interactions (haptics) are the only mode of communication between the subjects, in addition to them sharing common visual input (both see the screen where their actions are enacted). Any strategy, simple of complex, would have to emerge from the individuals negotiating their actions using that information. Although it is plausible that in some very specific scenarios, simple strategies could indeed emerge that could be described in simple cognitively comprehensible rules (e.g., this could be the case when the coins form some regular pattern), in our experiments this was not the case.

**Figure 8:**
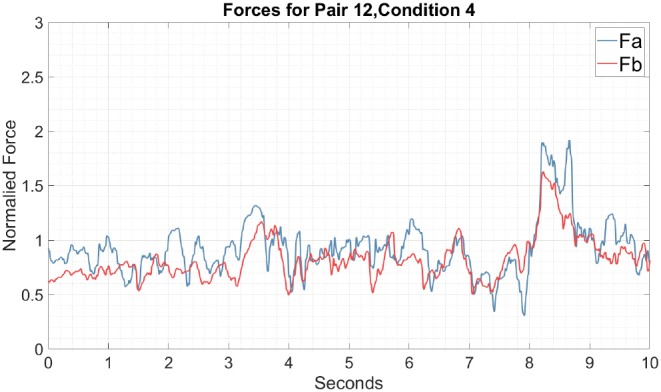
Typical example of the force as a function of time for Pair 12 in the one-coin condition. The blue trace represents the force recorded from participant A and the red trace represents the forces recorded from participant B. Both forces are in the horizontal direction. Condition 1 is the simplest environment for paired participants given that only one target was presented at a time. The force time series also shows an overlap of forces during the experiment, which again suggests that participants shared control of the device. If participants had distributed control over the device it would be visible in the time series as alternating peaks.

Now, focusing on the horizontal movements measured by the force sensors at both sides of the cylinder, Figure 8 shows both positively and negatively correlated forces as a function of time. In the following sections, we will focus on the mutual interactions based on the horizontal movements in order to characterize the temporal correlations of the haptic signals.

**Figure 9:**
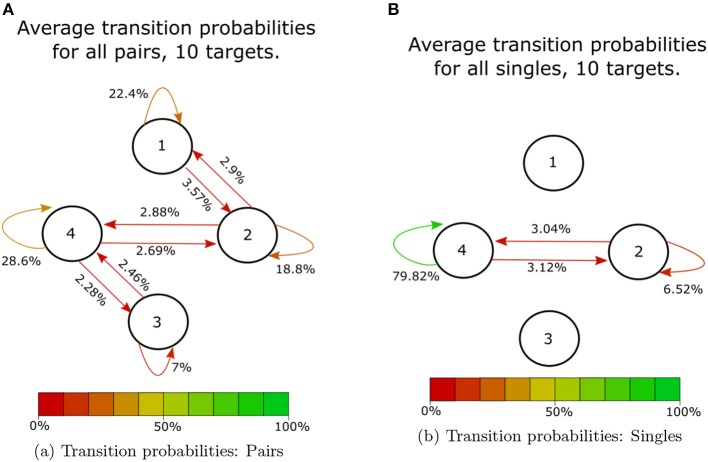
Average transition probabilities over all 10 coin trials. Each node corresponds to one of the haptic states identified above. **(A)** Shows average transition probabilities for all 14 paired participants. **(B)** Shows average transition probabilities for all 28 single participants.

**Figure 10:**
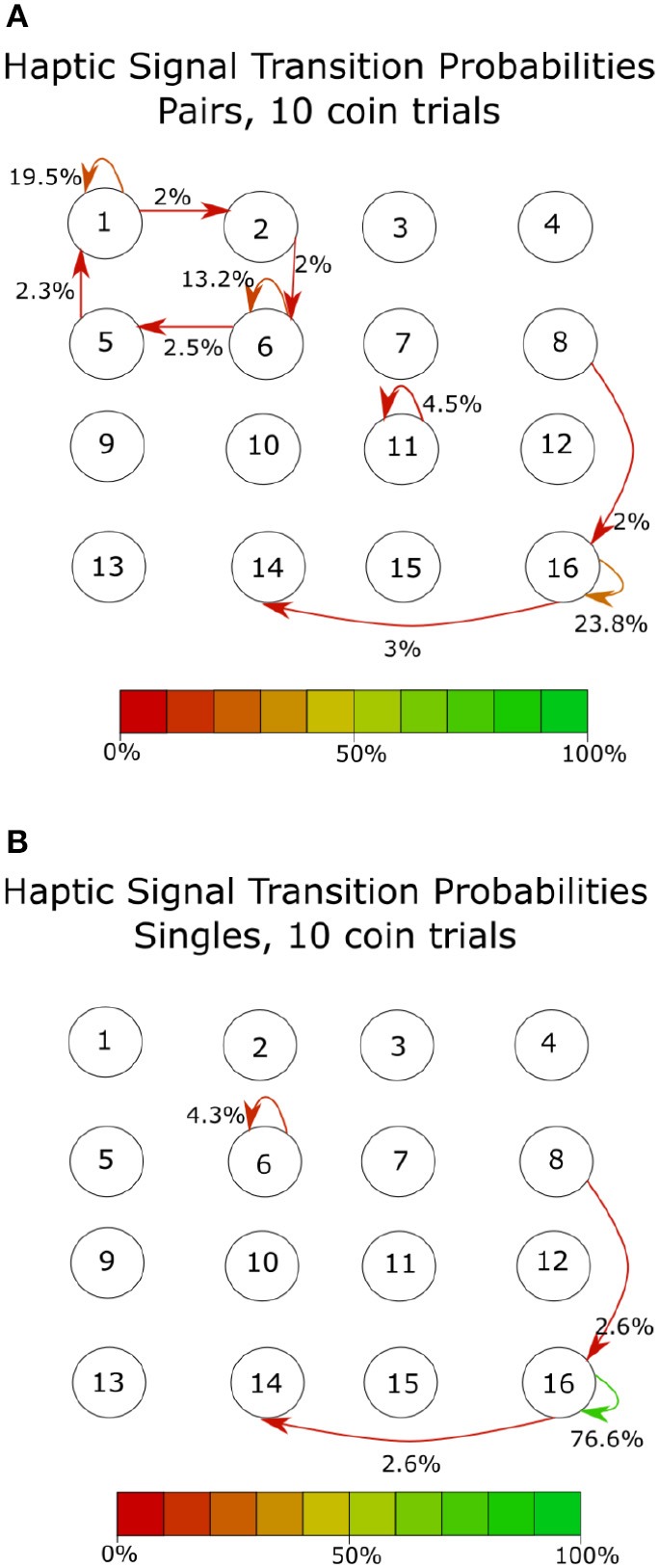
Transition probabilities over all participants for 16 haptic signals, defined in this case as two consecutive haptic states. The row number in this figure represents the state at t = 1 and the column number represents the state at t = 2. For example, haptic signal 6 represents haptic state 2 at t = 1 and haptic signal 2 at 2 = 2, a full list of the haptic signals and their corresponding states can be seen in Figure 2. The color of the arrows shows the transition probability from signal to signal. All transitions below 2% were discarded. **(A)** Shows the transition probabilities between signals during the paired participants condition. **(B)** Shows the transition probabilities between signals during the single participant condition. The symmetry shown by paired participants in states s1 and s2 suggests that participants shared control of the device in order to collect as many coins as possible when taking all trials into account. Paired participants used a wide array of signals, therefore increasing the potential for communication while single participants have no need to communicate and are possibly only using forces as a means of stabilizing the device. Data shown here was averaged over all 28 single participants and 14 paired participants.

At the shortest time scale, we looked at participants' immediate responses to incoming haptic states. Figure 9 shows the average transition probabilities for all paired participants, during 10-coin trials, between haptic state. The results indicate that single participants spend most of their time applying above-threshold forces to both sides of the cylinder, which we have labeled as state 4. This behavior allowed single participants to stabilize the cylinder and move quickly from target to target. On the other hand, paired participants can be in any of the four haptic states. State 1 corresponds to a fully receptive state where neither participant is applying an above-threshold force on the device, states 2 and 3 correspond to unilateral receptive states where either *F*_*a*_ of *F*_*b*_ is above the threshold and the other is below the threshold. By averaging over all paired participants, we found that the transitions between states were all roughly equal to 3% except transitions between states 1 to 3 and 2 to 3. We also found that participant B was in a receptive state twice as much as participant A. This is reflected in the difference in the stability of states 2 and 3, shown in Figure 9. Single participants, on the other hand, had a very stable state, 4, where they spend about 80% of their time. This lack in variation suggests that there is no communication taking place in the single participant condition. The transition probabilities of the haptic signals also show a similar pattern Figure 10. There is a clear lack of variation in the signals used by single participants. Paired participants show a more varied collection of symbols. Particularly in the loop between signals 1,2,5 and 6.

**Figure 11:**
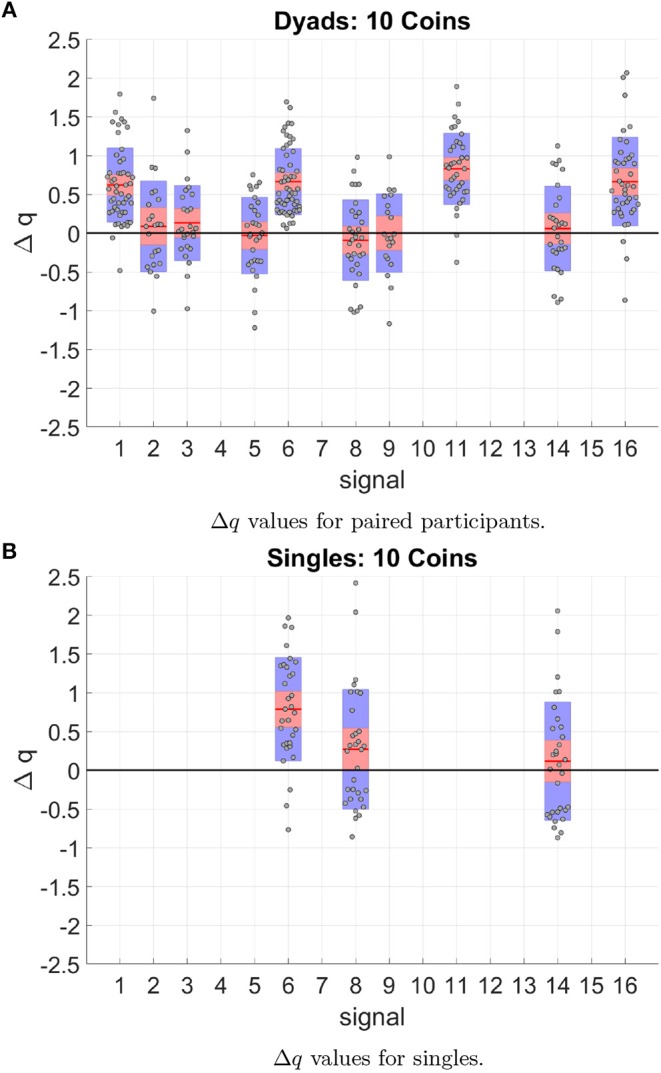
Δ*q* values for all 10 coin trials and over all participants. Each data point corresponds to one trial of one participant, the red line shows the average of all data for a particular signal. The red outline shows the 95% confidence interval of the average value and the blue outline shows the standard deviation. Signals that appeared less than 5 trials over all participants were removed. **(A)** Shows the Δq values for paired participants. **(B)** Shows the Δq values for single participants. Although both paired participants and single participants showed temporal correlations which are longer and shorter than a random distribution, only paired participants have long-range temporal correlations in signals which include a receptive state (signals 1–5). Single participants only showed long-range temporal correlations for signals where there is pressure being put on both sides of the device and for signal 6, participants only applied force on Fa. It is important to also note that only 30% of the distance distributions of identified haptic signals could be fit by the q-exponential in single-participant experiments. In paired participants this percentage increased to 80%; this suggests that signals recorded between paired participants have a mixture of long- and short-range temporal correlations. Data shown was averaged over all 28 single participants and 14 paired participants.

To further understand the exchange of information taking place during mutual haptic interactions, it was necessary to analyze the long and short-range temporal correlations present in the haptic signal time series. By calculating the temporal correlations of each possible haptic signal, we were able to identify key behavioral differences between the paired participants and the single participants the Δq values for each signal are shown in Figure 11.

When calculating the q-values for paired participants we found that 80% of the signals found in the data could be fit by the q-exponential. The distributions that could not be fit by the q-exponential function corresponded to signals with only short-range correlations. In the case of single participants, only 30% of the signals identified in the time series had any long-range correlations.

More importantly, signals found between paired participants that were not found in single participant trials are ones where one state is purely receptive or unilaterally receptive. One example of a combination that meets these requirements is signal 2 which is a combination of S_1_ = 1, S_2_ = 2. Figure 11A shows a clear connection between signals with receptive states (1,2, 5 and 6) that is missing for single participants (Figure 11B). These signals are a binary pulse that participants in paired participants could be using to guide their partner to the next target. This suggests that combinations of purely receptive states and unilateral receptive states were being used as a means of transferring information between paired participants.

In this study, we used non-extensive entropy to characterize the distribution of long- and short-range temporal correlations of what we have defined as haptic signals. By testing different lengths of haptic signals we found that signals with a length of two haptic states, on average, showed the largest non-extensivity values. This shows that signals made up of two haptic states have the most long-range temporal correlations and are the best candidates for being a means of communication. By analyzing the temporal correlations in the haptic signals, we were able to identify temporal structures similar to those found in other forms of coding which transfer information, such as genetic nucleotides and in human language (Mehri and Darooneh, 2011; Moghaddasi et al., 2017). In particular, the combination of long- and short-range temporal correlations has been shown to be a key feature of the emergence of proto-languages (Uno et al., 2011).

## 5. General Discussion

In summary, we were able to find behavioral characteristics unique to single and paired participants. Single participants spent most of their time applying pressure to both sides of the device, state 4 (Figure 9). This results in a very low percentage of the identified haptic signals having any long-range correlations. Paired participants showed a variety of possible haptic signals, most of which included a receptive haptic state (Figures 9, 10A).

Results show that the haptic signals used by paired participants have both long and short-range temporal correlations as indicated by the larger Δ*q* values and a larger proportion of signals that could be fit by the q-exponential function as shown in Figure 11. This result indicates the rich nature of the temporal patterns unique to haptic interactions between paired participants as a result of the real-time coupling of the action-perception loops.

The combination of longer and shorter-range temporal correlations suggests that the forces applied to either side of the signal are indeed being used as a means of transmitting information between the paired participants.

The temporal patterns of haptic signals during haptic interactions should be a result of a generated internal model of the partner. This model simulates the response of the partner in order to coordinate one's next motion in harmony with the partner.

In past literature, we find three main computational models that try and explain how groups of participants are able to coordinate their movement: (1) The “no computation” model proposes that paired participants follow their target independently. (2) The “follow the better partner” model supposes that haptic information allows members of the dyad to judge their partner's performance. If the partner is better than them then they switch to following the partner. (3) The “multisensory integration” model assumes that the haptic forces allow partners to estimate the other's position and track a weighted combination of this estimate and the target depending on how reliable the information is Takagi et al. (2017). Takagi et al. proposed a fourth model that, as they showed, could accurately reproduce experimental observations. They proposed that partners used the haptic forces to estimate their partner's target and to improve their prediction of the target's movements. It is important to note that during these experiments participants had some haptic information from their partner but not enough to influence their movements or to let them know that they were haptically coupled to another person. The participants were also only tracking a single target which was moving in a circular trajectory at a constant speed during each trial.

In the context of control engineering, the internal model is often identified as the feed-forward model in a control loop. Takagi et al. showed that paired participants could identify a common target using only the mutual force-force interactions (Takagi et al., 2017), and they suggested that this ability is due to the creation of a feed-forward model which can not only be used to predict the outcome of one's own movements but also to predict the outcome of a partner's movements. The same principles must also be present in the “joint coin-collecting” paradigm in this study. The paired participants need to build an internal model of the partner to simulate the motion of the partner.

It is important to note a key difference between Takagi's experiment and the “joint coin collecting” paradigm. The joint coin-collecting paradigm is a truly cooperative task: both participants jointly control a single cursor and are rigidly coupled to one another through moving a rigid rod. In Takagi's experiment, participants are unaware of each other, and in principle, both participants are engaged in doing a reaching task on an individual basis, even though their hands are weakly connected by a virtual spring, and the interaction force is so small that the participants would not notice the existence of a partner.

The emergence of the long- and short-range temporal correlation might indicate a proto-language emerging from the force-force interactions. Signals combining purely receptive states and unilateral receptive states show both larger q values, which are associated with a mixture of long- and short-range temporal correlations which have both been found to be essential for the emergence of proto-languages (Uno et al., 2011).

Understanding the temporal structure of haptic interactions in humans can lead us to implement the same algorithms to Human-Machine Interfaces. For example, by maintaining the temporal structure of the haptic interactions, haptic guidance can be provided to humans to achieve a demanding task in motor coordination. We anticipate that humans could feel as comfortable and secured as if they were interacting with a human partner. In a future study, we will develop an artificial agent which can provide the haptic guidance to human partners and study if the human-like interactions can produce better skill transfer in motor coordination.

## Data Availability Statement

The datasets generated for this study are available on request to the corresponding author.

## Ethics Statement

The studies involving human participants were reviewed and approved by University of Reading Systems Engineering Department and University of Reading Psychology Department. The patients/participants provided their written informed consent to participate in this study.

## Author Contributions

NT, YH, and SN conceived, designed the experiments, and wrote the paper. NT performed the experiments and analyzed the data. YH, SN, JH, and TK provided guidance and edited the paper. All authors reviewed the manuscript.

### Conflict of Interest

The authors declare that the research was conducted in the absence of any commercial or financial relationships that could be construed as a potential conflict of interest.
