# Supplementary figures and images for: Temporal Structure in Haptic Signaling Under a Cooperative Task
Source: Front Hum Neurosci. 2019 Nov 27;13:372. doi: 10.3389/fnhum.2019.00372 (PMC6890600; doi:10.3389/fnhum.2019.00372)

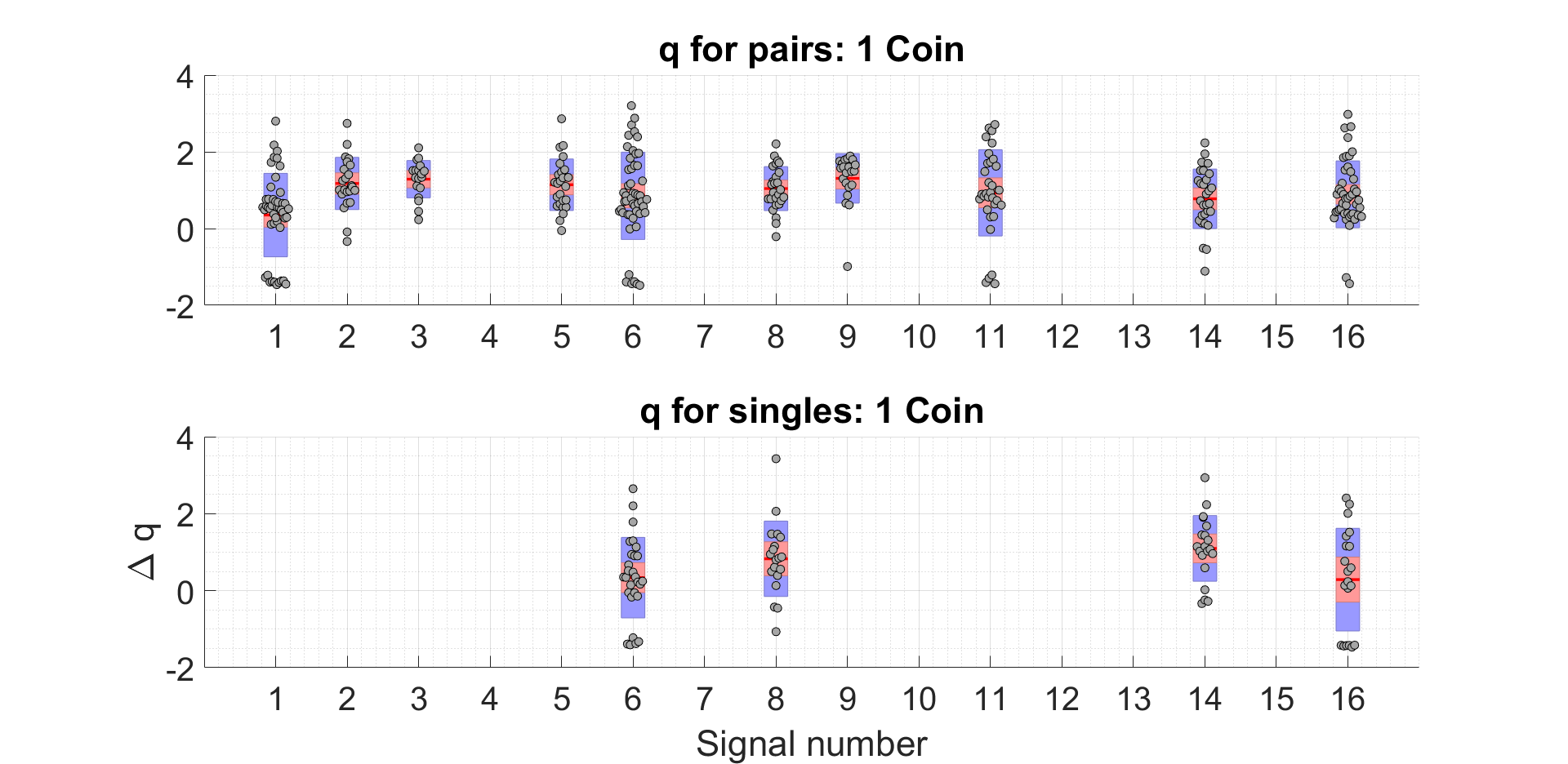

Supplement: Supplementary file 1 [file Data_Sheet_1.zip › AppendixHaptics/qCond1.tif]

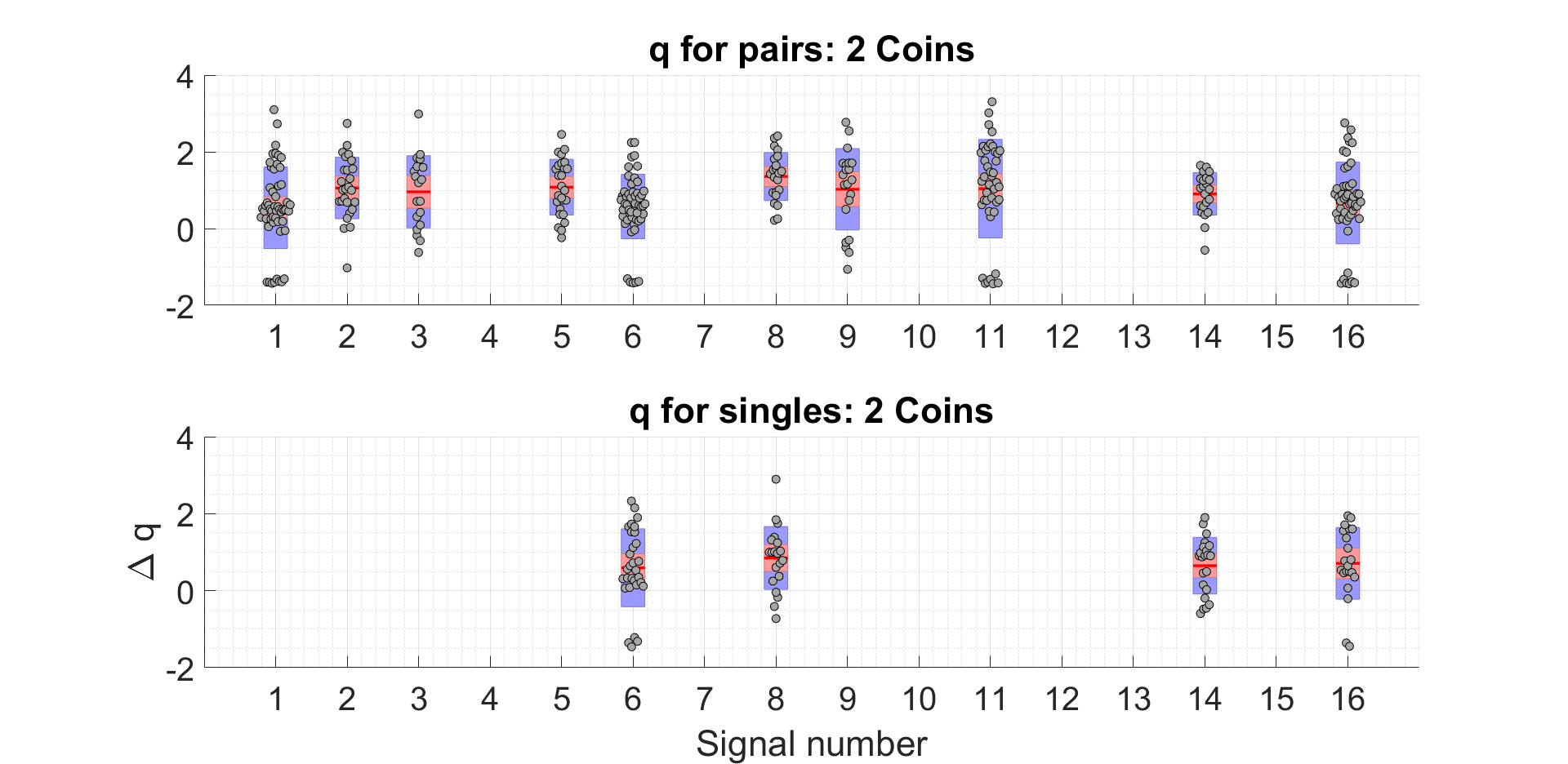

Supplement: Supplementary file 1 [file Data_Sheet_1.zip › AppendixHaptics/qCond2.tif]

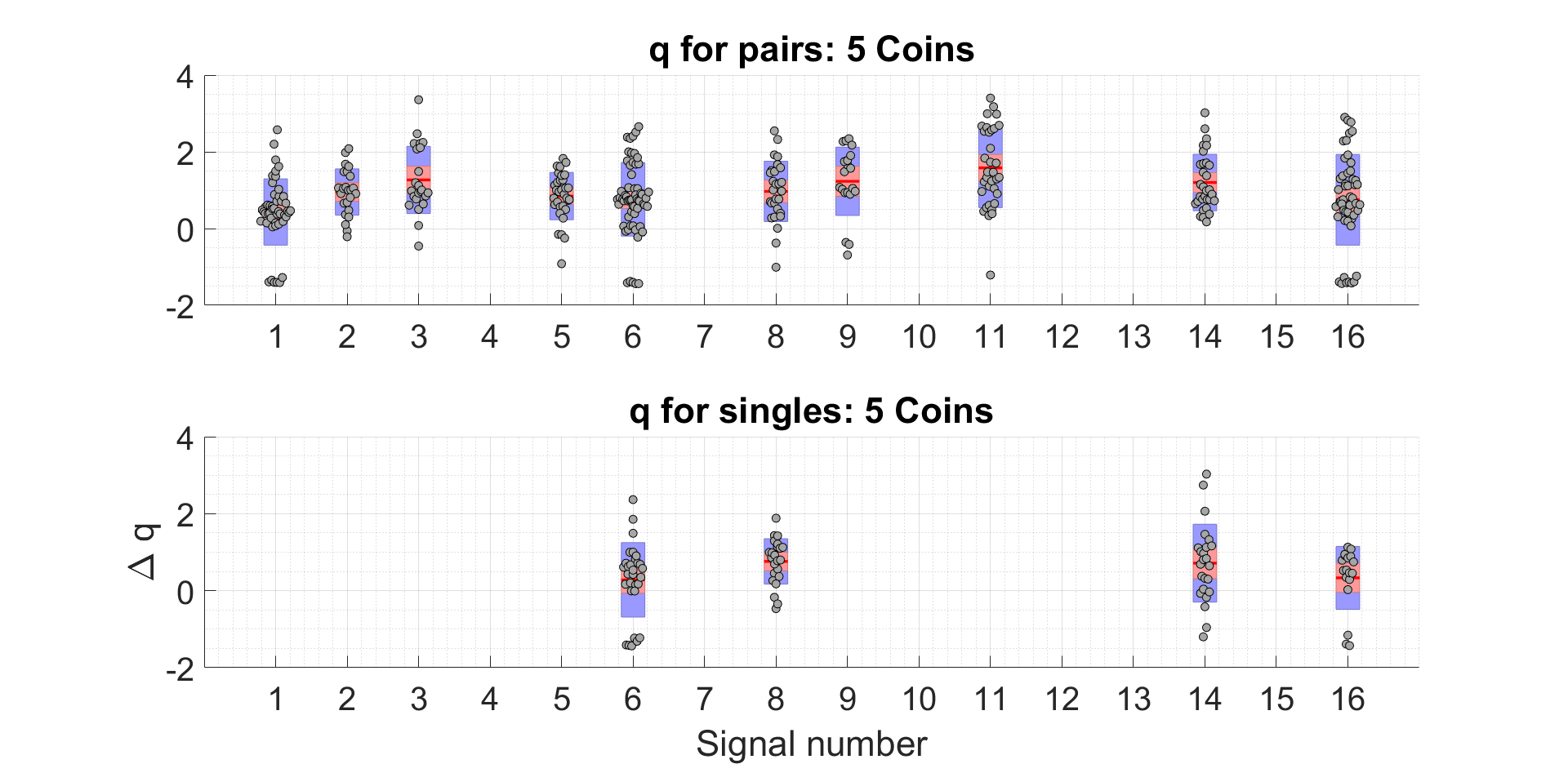

Supplement: Supplementary file 1 [file Data_Sheet_1.zip › AppendixHaptics/qCond3.tif]
